# Is Conservative Treatment Superior to Surgical Intervention in Hematogenous Primary Septic Spinal Infection in Terms of Mortality, Recurrence, and Hospital Stay? A Systematic Review and Meta-Analysis

**DOI:** 10.3390/jcm14248650

**Published:** 2025-12-06

**Authors:** Panagiotis Korovessis, Vasileios Syrimpeis, Georgios Dimakopoulos

**Affiliations:** 1 Orthopedics Department, Olympion General Clinic, 26443 Patras, Greece; vsyrimpeis@gmail.com; 2 Science and Technology Park of Epirus, University Campus of Ioannina, 45500 Ioannina, Greece; info@biostats.gr

**Keywords:** spinal infection, spondylodiscitis, discitis, osteomyelitis, spondylitis

## Abstract

**Background/Objectives:** The treatment of Hematogenous Primary Septic Spinal Infection (HPSSI) typically involves either conservative management or surgical intervention. Previous studies have suggested that conservative treatment with antibiotics is the mainstay conventional procedure in treating HPSSI, but relevant conclusions remain controversial regarding mortality, recurrence, and hospital stay. There is a lack of systematic reviews and meta-analyses comparing conservative vs. surgical treatment specifically in non-TBC, non-fungal, and non-postsurgical HPSSI in adults. This systematic review and meta-analysis aim to systematically evaluate the therapeutic effectiveness of conservative versus surgical intervention for the management of HPSSI, through a meta-analysis of key outcomes including mortality, recurrence, and length of hospital stay. **Methods:** A comprehensive literature search was performed across four major databases: PubMed, Cochrane, Science Direct, and Scopus. Using defined inclusion and exclusion criteria, we identified twelve studies encompassing 1199 patients with hematogenous, primary, septic spinal infection, which was not post-surgical, not due to TBC, and not fungal, who were treated conservatively (*n* = 519) or surgically (*n* = 680) for inclusion in the meta-analysis. PRISMA guidelines were used for this analysis. The primary outcome analyzed was mortality; secondary outcomes were infection recurrence and length of hospital stay, comparing conservative treatment versus operative intervention for HPSSI. **Results:** Mortality rates for surgical versus conservative treatment varied across five studies. Some studies reported significantly lower mortality with surgical intervention vs. conservative treatment (2.3–6% vs. 17.8–18%), while others showed no difference (11% for both treatments). This meta-analysis indicates that surgical treatment does not significantly alter mortality rates compared to conservative management, although study heterogeneity is considerable. Infection recurrence was reported in three studies, with rates ranging from 5 to 16.4% for conservative treatment and 5 to 11.6% for surgical intervention. These differences were not statistically significant in studies that provided group-specific data. Findings on hospital length of stay were mixed: two studies reported shorter stays for surgical patients (23.9–33.4 days vs. 40.5–51.2 days), while another study found no meaningful difference between the groups. Across multiple studies, advanced age, frailty, higher comorbidity burden, and neurological impairment were consistently identified as independent predictors of increased mortality, irrespective of treatment modality. Although some data suggest a short-term survival advantage associated with surgical intervention, the overall mortality outcomes remain heterogeneous across the literature. **Conclusions:** Overall, the findings of this meta-analysis remain inconclusive regarding which treatment—surgical or conservative—is more advantageous in reducing mortality, infection recurrence, and hospital stay. The variability across studies highlights the influence of patient selection, treatment protocols, and local clinical practices. To enhance our understanding and improve outcomes in HPSSI, future randomized controlled trials are essential. These studies should incorporate clear selection criteria, standardized terminology for spinal infection subgroups, and homogenous patient populations with well-defined comorbidities to allow for meaningful data comparisons. Additionally, emphasis should be placed on early diagnosis, rapid identification of causative pathogens, using modern diagnostic tools, and timely initiation of appropriate treatment—whether surgical or conservative—to optimize patient outcomes, including reduced mortality, lower recurrence rates, and shorter hospitalizations.

## 1. Introduction

Hematogenous Primary Septic Spinal Infection (HPSSI) represents a spectrum of primary bacterial infections (not post-surgical) involving the vertebral column, e.g., vertebral osteomyelitis and spondylodiscitis, alone or associated with spinal epidural abscess. Although relatively uncommon—accounting for 2–7% of musculoskeletal infections with an annual incidence of 0.2–2 per 100,000—HPSSI carries a high risk of morbidity and mortality, particularly in elderly or immunocompromised patients. Staphylococcus aureus remains the predominant pathogen, responsible for up to 80% of cases [1,2,3,4,5,6,7,8].

Spinal infections, though relatively rare, represent a significant clinical challenge that often necessitates prompt and intensive medical and sometimes surgical intervention. HPSSI is a serious disease associated with potentially high mortality, taking its highest value in the early stage following diagnosis [9,10,11,12,13,14,15].

Spondylodiscitis is the most common HPSSI subgroup and affects mainly elderly immune-compromised patients; its estimated mortality rate ranges between 2% and 10% [5,16,17,18,19,20].

Diagnosis is typically achieved through image-guided or intraoperative aspiration or biopsy of the disk space and/or vertebral endplate, allowing for both microbiological and pathological evaluation [14,15,21]. Definitive treatment should be guided by culture results and antimicrobial susceptibility testing. Recent literature cites age and comorbidities as being vital in deciding optimal treatment strategies [22,23]. Generally, the success of the treatment is evaluated by clinical monitoring, along with laboratory markers like C-reactive protein and erythrocyte sedimentation rate, and it is traditionally sufficient to assess response to treatment [24].

The most severe complications associated with HPSSI include sepsis, segmental spinal instability and neurological impairment, formation of epidural and subdural abscesses, meningitis, loss of cervical or lumbar lordosis, and collapse of vertebral segments, potentially resulting in progressive spinal deformity [25,26,27,28,29,30,31,32,33].

The current standard of care for HPSSI is a conservative form of treatment that consists of the administration of antibiotics, bed rest, and bracing; however, if the HPSSI is complicated (intractable spinal pain, neurologic impairment, and/or abscess formation), surgical treatment is considered [10,34]. Conservative treatment succeeds in around 80–90% of well-selected cases (i.e., early disease, no neurological deficits, manageable comorbidities) [35]. Studies report failure rates for conservative management in the range of 12–16%, where progression of infection and complications need surgical intervention [36].

Surgical treatment is indicated in those patients who have a poor response to conservative treatment, vertebral body collapse and/or spinal deformity due to spinal infection, or have a neurological deficit, possibly associated with spinal epidural abscess compression on the spinal cord or disease progression despite appropriate antibiotic treatment [4,37,38,39,40,41,42,43,44,45,46].

Decompression of neural elements by laminectomy, laminotomy, or debridement with/without spinal instrumentation for HPSSI has been described [47].

Recurrence rates of HPSSI vary widely, ranging from 2% to 9.9% [27,48,49]. The overall mortality of HPSSI has been reported to be up to about 20–28% and appears to be particularly high in the first 3 months after diagnosis settlement [9,10,11,12,13,15,50,51].

Several studies have identified factors associated with poor outcomes and short-term mortality in patients with HPSSI, including advanced age, comorbidities, severe neurological deficits on admission, rheumatoid arthritis, elevated serum creatinine and C-reactive protein levels, and the presence of *Staphylococcus aureus* infection [9,52]. Prompt and aggressive treatment of central line-associated bloodstream infections (CLABSIs) has been shown to reduce the incidence of vertebral paraspinal infections [53].

The diagnosis of HPSSI is often delayed because of its insidious onset and nonspecific clinical presentation. Microbiological confirmation is essential for establishing the diagnosis of hematogenous pyogenic spinal infection (HPSSI) and for guiding targeted antimicrobial therapy. Culture-based identification of the causative pathogen enables spine surgeons to tailor antibiotic treatment, monitor therapeutic response, and avoid unnecessary use of broad-spectrum or prolonged empirical regimens. Specifically, **blood cultures** are positive in approximately 40–70% of HPSSI cases with evident bacteremia, with the highest yield achieved when samples are obtained before initiation of antibiotic therapy [1,9,35]. In some series, blood cultures alone have established the diagnosis in up to two-thirds of patients. **Image-guided needle biopsy** (CT- or MRI-guided) demonstrates a diagnostic yield ranging from 40 to 80%, depending on factors such as technique, timing, and prior antibiotic exposure. Repeating the biopsy can increase diagnostic success, particularly when the initial attempt is non-diagnostic. Notably, the yield decreases substantially if performed after antibiotic initiation (reported reductions from 76% to 45%) [1,9,35]. Finally, **intraoperative cultures** obtained during open biopsy or surgical debridement offer the highest microbiological yield—typically exceeding 90%—especially in cases requiring decompression or stabilization. This approach also allows for simultaneous histopathological confirmation and therapeutic debridement [1,9,35].

Besides blood cultures, advanced imaging and microbiological confirmation via image-guided or intraoperative biopsy are critical for diagnosis, yet false-negative results and delays in culture growth are frequent. Early recognition is essential, as untreated infection can lead to severe complications, including epidural abscess formation, sepsis, spinal instability, and irreversible neurological deficits. In the case of negative blood or biopsy/cultures, repeated blood and biopsies are recommended. Microbiological confirmation is essential for establishing the diagnosis of HPSSI and guiding targeted antimicrobial therapy. Culture-based identification of the causative pathogen allows spine surgeons to tailor antibiotics, monitor treatment response, and avoid unnecessary broad-spectrum or prolonged empirical regimens.

The optimal management of HPSSI remains controversial. Standard therapy consists of targeted antimicrobial treatment with immobilization and supportive care, achieving success in most early or uncomplicated cases. However, surgical intervention is indicated for patients with neurological compromise, spinal deformity, abscess formation, or treatment failure. Reported mortality rates range from 20% to 28%, and conservative treatment failure has been described in up to 15% of patients. Conversely, surgery is associated with procedure-related risks in sick, elderly patients with associated comorbidities and variable functional outcomes, leaving uncertainty about the relative benefits of early surgical intervention versus medical therapy alone.

The existing literature demonstrates substantial heterogeneity in study design, patient selection, and outcome definitions, leading to inconsistent conclusions regarding mortality, recurrence, and hospitalization length. High-quality comparative evidence is limited, and most studies are small retrospective cohorts.

Given these uncertainties, a comprehensive synthesis of available data is needed to clarify the relative outcomes of surgical versus conservative management in adult patients with bacterial, non-tuberculous, non-fungal, and non-postoperative HPSSI. The present meta-analysis aims to compare these treatment strategies with respect to mortality, infection recurrence, and hospital length of stay, thereby informing evidence-based clinical decision-making.

## 2. Materials and Methods

### 2.1. Literature Search

**Figure 1:**
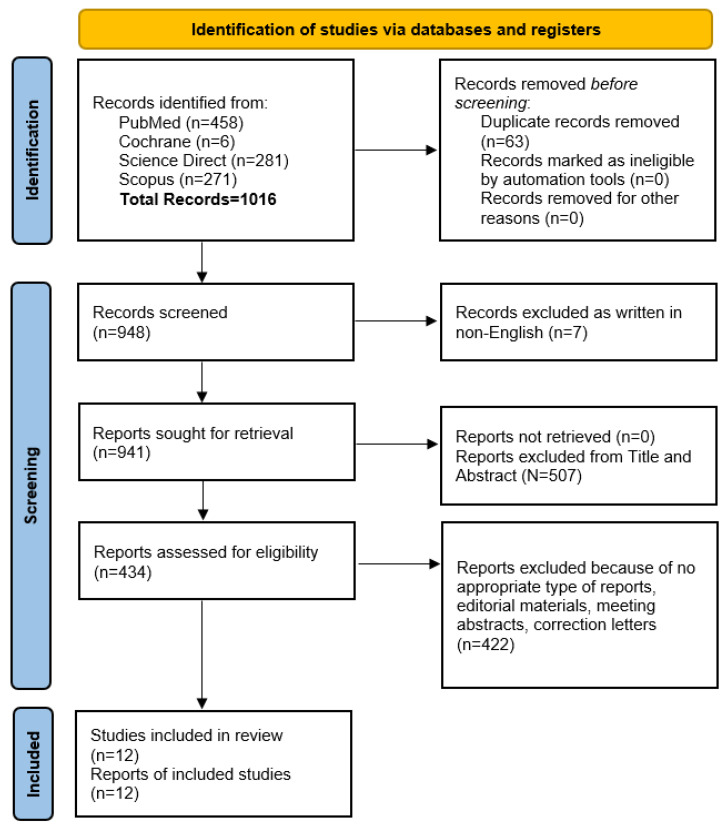
PRISMA flowchart for the systematic review and meta-analysis.

Following PRISMA (Preferred Reporting Items for Systematic Reviews and Meta-Analyses) guidelines [54], a systematic search was conducted across **PubMed, Cochrane, ScienceDirect,** and **Scopus** to identify studies published between 2005 and 2025 (see Figure 1 and File S4 PRISMA Checklist). The process was designed to identify all eligible studies using the following queries:

1. (“spondylodiscitis” OR “spondylitis”) AND NOT “ankylosing spondylitis” AND NOT “tuberculosis”.
2. (“spinal infection” OR “infection of the spine”) AND (“conservative treatment” OR “surgical treatment”).

No language filters were used during the search process, and the date of the last search was 30 March 2025.

The articles included in this review met the following criteria:

1. **Study Design**: Retrospective double-arm cohort studies comparing surgical to conservative treatment of spinal infections. No RCTs were found in our search.
2. **Comparative Analysis**: Studies comparing surgical treatment (ST) to conservative treatment (CT).
3. **Patient Population**: Studies involving patients with hematogenous, septic spondylodiscitis or spondylitis—not ankylosing spondylitis, not tuberculosis, not post-surgical, and not fungal.
4. **Primary Endpoint: Studies with the primary endpoint of** the incidence of hematogenous spinal infection.

In cases where duplicate or overlapping data were identified in multiple studies, only the study with the most complete and comprehensive data was included in the review.

The criteria for including studies in this review were based on the **PICO [55]** (Population, Intervention, Comparison, and Outcome) framework.

1. **Population (P): Adults with primary hematogenous septic spinal infection**, encompassing the subgroups of **native spondylitis, septic spondylodiscitis, discitis, spondylitis**, and **vertebral osteomyelitis**, specifically referring to adult patients (≥18 years) diagnosed with an acute or subacute infection of the spinal column resulting from **hematogenous spread of pathogens**, primarily **bacterial** in origin, affecting the **cervical, thoracic, thoracolumbar, lumbar, or lumbosacral spine**.
2. **Interventional Treatment (I)**: Traditional open anterior, posterior, or combined surgery, using minimally invasive, endoscopic techniques potentially involving spinal instrumentation.
3. **Comparison Controls (C)**: Patients who received conservative treatment (CT) (antibiotics, brace, aspiration).
4. **Outcome (O)**: Incidence rate differences between conservative and surgical treatment regarding **mortality, recurrence, and length of hospital stay**.

The **inclusion criteria** for the meta-analysis were as follows:

- **Condition**: Adults with hematogenous, septic spondylodiscitis or spondylitis—not ankylosing spondylitis, not tuberculosis, not fungal, and not caused by previous surgery or trauma.
- **Clinical Presentation**: Fever, pain, or cachexia.
- **Diagnostic Confirmation**: MRI showing destruction of the vertebral endplates and disks, roentgenograms, biopsy, or elevated CRP.
- **Study Size**: Studies with at least 10 cases each.
- **Outcome Reporting**: Series specifically reporting on adults with hematogenous, septic spondylodiscitis or spondylitis—not ankylosing spondylitis, not tuberculosis, not fungal, and not caused by previous surgery or trauma—treated surgically, conservatively, or both.
- **Language**: Articles published in English and German.

The **exclusion criteria** for the meta-analysis were as follows:

- **Study Type**: Narrative reviews, systematic reviews, meta-analyses, and case reports; studies reporting solely or mainly on spinal abscesses.
- **Sample Size**: Studies with fewer than 10 participants.
- **Language**: Articles published in languages other than English and German.
- **Intervention**: Studies that identified spinal intracanal or paravertebral abscess as the main focus of investigation.
- **Study Population**: Studies including spinal infections due to TBC, mycosis, abscess, or post-surgical spinal infections.

From each eligible article, the following information was extracted and documented in an Excel sheet: (1) the authors’ name; (2) year of publication; (3) type of study; (4) demographic and baseline characteristics of participants; (5) type of conservative treatment; (6) type of surgical treatment; (7) follow-up details; (8) outcome data (mortality in conservative and surgically treated patients, recurrence, reoperation rates, hospital stay); (9) reported complications, etc.

### 2.2. Selection of Studies

The search process was blinded, with only article titles and abstracts reviewed initially. Two independent observers assessed the quality and bias of the retrieved studies independently. Articles were selected based on the inclusion criteria to minimize bias in both study selection and data extraction.

### 2.3. Key Steps in the Review Process

- ***Initial Selection***: Articles were evaluated based on titles and abstracts. Full texts were retrieved for studies with unclear inclusion/exclusion criteria.
- ***Resolution of Doubts***: If uncertainties persisted, the decision was made through discussion, with a third reviewer brought in if necessary.
- ***Scoring***: Each primary study was assigned a score independently by the two reviewers. The final score for each study was the average of the two scores.

### 2.4. Protocol and Data Extraction

A written study protocol was developed before starting the literature review, including clearly defined eligibility criteria. Two investigators independently extracted relevant data from each trial using a standardized form. The same observers independently extracted data from each article to ensure consistency. This rigorous approach ensured a systematic and unbiased review process. An overview of the common characteristics of the 12 studies finally included in the analysis is shown in Table 1. The protocol for this meta-analysis has been registered in the PROSPERO database: CRD420251065141.

The data supporting this meta-analysis have been deposited in the Mendeley Data repository and can be accessed at https://data.mendeley.com/datasets/4w88x9t75k/1 (accessed on 14 October 2025).

A total of twelve retrospective cohort studies (see Table 1) were included in this review. All studies adopted a comparative design, evaluating at least two different treatment strategies.

With respect to the underlying condition, the majority of studies (*n* = 9) focused on pyogenic spinal infections, including spondylodiscitis, vertebral osteomyelitis, spondylitis, spinal infection, and spondylodiskitis. The three remaining studies investigated specific infection types: one on vertebral osteomyelitis that was not otherwise specified, one on *Staphylococcus aureus* vertebral osteomyelitis, and one on osteomyelitis/discitis.

All studies compared surgical to non-surgical treatment approaches. Eight (8) studies contrasted surgical interventions of various types with conservative management, which included antibiotics, bed rest, bracing, or orthosis. Two (2) studies directly compared surgery with medical management consisting of antibiotics alone. One study examined early surgery combined with antibiotics versus antibiotics alone, while another one compared surgical debridement and stabilization with antibiotic therapy. Notably, no studies were identified that exclusively compared non-surgical interventions or different surgical techniques.

The **primary outcome** analyzed is mortality; secondary outcomes are infection recurrence and length of hospital stay, comparing conservative treatment versus surgical treatment for HPSSI.

### 2.5. Quality Assessment and Risk of Bias

The meta-analysis was conducted in accordance with the recommendations of the Cochrane Collaboration and the guidelines for Quality of Reporting of Meta-analyses [55,59,60,61]. The analysis of the outcomes was divided into subgroups according to surgical (ST) or conservative treatment (CT). The retrospective studies’ quality was justified using the Newcastle‒Ottawa scale (NOS) [62] (see Table 2). The NOS includes the following three areas—(1) patient representation, (2) exposure and outcome determination, and (3) follow-up adequacy—with a maximum total score of 9 for each study. NOS scores of 0–5, 6–7, and 8–9 indicate low, moderate, and high quality, respectively. A ROBINS-I assessment for non-randomized comparative studies with domain-level judgments was also performed and uploaded as Supplementary Materials File S1.

The possibility of publication bias was assessed by analyzing a funnel plot using RevMan 5.4. Symmetry in the funnel plot suggests the absence of publication bias, whereas asymmetry may indicate the non-publication of small trials with negative results or a preference for publishing studies with favorable outcomes.

### 2.6. Statistical Analysis

Heterogeneity across the studies was assessed using the chi-square test and I^2^ statistic, with I^2^ values ranging from 0 to 100% indicating low, moderate, and high heterogeneity at values of 25%, 50%, and 75%, respectively.

The analysis was carried out for retrospective design studies and aimed to estimate the pooled effect size. We extracted the odds ratios (ORs) to describe the outcomes of interest data, with 95% confidence intervals (CIs). The pooled OR or MD was estimated for the retrospective studies. Continuity correction was applied in studies with zero cell counts. The conceptual background of the included studies indicated that all estimates should be based on random-effects models and the inverse variance method. In this meta-analysis, the pooled estimate was derived under a random-effects model using the inverse variance method [63]. A sensitivity analysis was conducted to assess the impact of individual studies on the overall inference. In addition, a sensitivity analysis was performed to assess the influence of studies that did not adjust for potential confounders. These unadjusted studies were excluded in a secondary analysis to determine whether their inclusion biased the pooled effect estimates. No significant changes were observed in any case.

## 3. Results

**Figure 2:**
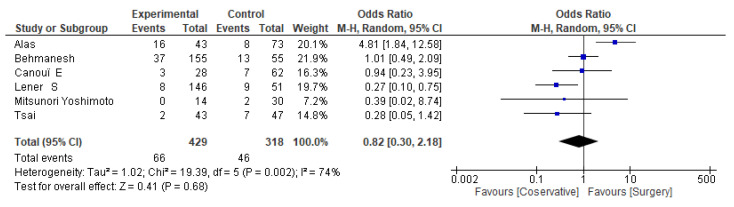
Mortality forest plot [26,30,31,32,33,56].

Regarding **mortality**, in this meta-analysis, individual study odds ratios (ORs) vary widely, with confidence intervals generally crossing 1.0, indicating non-significant differences for most studies. The overall odds ratio is 0.82 (95% CI: 0.30–2.18), indicating no statistically significant difference in mortality between surgical and conservative treatments. Heterogeneity is high (I^2^ = 74%), suggesting substantial variation between studies. The test for overall effect is non-significant (Z = 0.41, *p* = 0.68). See Figure 2 and Table 3.

**Figure 3:**
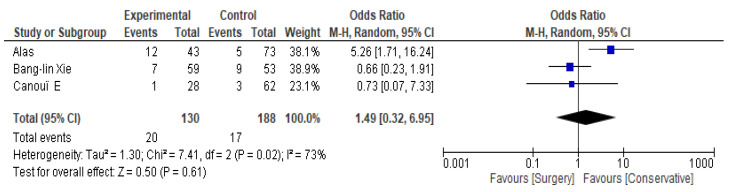
Recurrence forest plot [31,32,58].

The **incidence of infection recurrence** between surgical and conservative treatment was assessed in three out of the twelve included studies. The individual odds ratios (ORs) varied substantially, with confidence intervals crossing 1.0 in two of the three studies, indicating no statistically significant difference in recurrence rate. The overall odds ratio is 1.49 (95% CI: 0.32–6.95), indicating no statistically significant difference in recurrence between surgical and conservative treatments. Heterogeneity is high (I^2^ = 73%), suggesting substantial variation between studies. The test for overall effect is non-significant (Z = 0.50, *p* = 0.61). See Figure 3 and Table 4.

**Figure 4:**
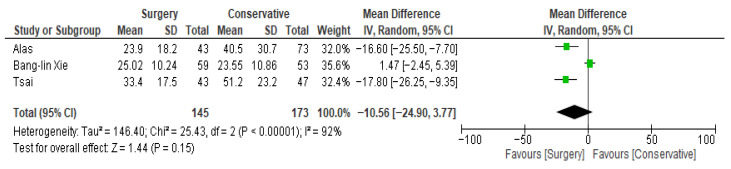
Hospital stay forest plot [32,33,58].

Regarding the **length of hospital stay,** differences between surgical and conservative treatment across the three studies showed that individual study odds ratios (ORs) vary widely, with confidence intervals crossing 0.0 in one of the three studies, indicating significant differences. The overall odds ratio is −10.56 (95% CI: −24.90, 3.97), indicating no statistically significant difference in the length of hospital stay between surgical and conservative treatments. Heterogeneity is high (I^2^ = 92%), suggesting substantial variation between studies. The test for overall effect is non-significant (Z = 1.44, *p* = 0.15). See Figure 4 and Table 5.

A GRADE Summary of Findings table for the three outcomes is also provided as Supplementary Materials File S2.

## 4. Discussion

Despite advances in diagnostic techniques and antimicrobial therapies, the insidious onset and nonspecific clinical manifestations of HPSSI often lead to delayed diagnosis and initiation of treatment, thereby contributing to suboptimal clinical outcomes.

HPSSI is frequently associated with serious complications, including sepsis, segmental spinal instability, and neurological compromise, as well as the formation of epidural and subdural abscesses. Additional sequelae may include loss of sagittal spinal alignment and segmental vertebral collapse, potentially leading to progressive spinal deformity.

In untreated patients, HPSSI is associated with a high overall mortality rate, reported to range between 20% and 28%, with the risk of being particularly elevated during the first three months following diagnosis. The mortality associated with HPSSI appears in higher levels in cases of septic spondylodiscitis with/without complications such as intraspinal, paravertebral abscess, and death, among others [9,10,11,12,13,15,50,51].

A notable inconsistency was observed across the included studies in our meta-analysis regarding **mortality**, with some reporting lower mortality rates in surgically treated patients, while others found no significant difference between surgical and conservative management. One study included in our review (Alas et al. [32]) reported a significantly lower 30-day mortality rate in the surgical cohort (2.3%) compared to the conservatively treated group (17.8%; *p* = 0.016)**.** Similarly, Lener et al. [26] observed an overall mortality rate of 6% in surgically managed patients versus 18% in those receiving conservative treatment (*p* = 0.017).

In contrast, Canouï et al. [31] found no difference in mortality between the two groups, with both reporting a rate of 11% (*p* = 1.00). Across the literature, increased age, frailty, comorbidity burden, and neurological impairment were consistently associated with elevated mortality, independent of management strategy. Our meta-analysis indicates that, based on the available data, surgical treatment does not significantly alter mortality compared to conservative management, although study heterogeneity is considerable. Thus, while some studies indicate a potential short-term mortality benefit associated with surgical intervention, the overall evidence remains heterogeneous and inconclusive across the broader body of data.

Our findings indicate that multiple patient-related comorbidities and disease-related factors significantly influence clinical outcomes in spinal infections, regardless of the treatment modality. Moreover, previous studies have demonstrated that spinal infections can affect various anatomical segments of the spinal column with differing frequencies, each potentially requiring a tailored therapeutic approach.

Consistent with previous literature, in 9 of the 12 studies in our meta-analysis, patients suffered from multiple serious comorbidities (Table 1). Comorbidities, as measured by the Charlson Comorbidity Index, frailty, and poor functional status were all linked to increased risk of death in three studies included in our meta-analysis [27,29,32], while advanced age was also associated with higher mortality in two studies [27,56]. In addition, the severity of infection—often indicated by neurological deficits, spinal instability, or failure of medical therapy—tended to select patients for surgical intervention, potentially confounding direct comparisons of mortality between treatment groups.

All twelve studies included in this meta-analysis were retrospective; no randomized controlled trials (RCTs) were identified in the searched literature addressing HPSSI. Nine of the twelve studies involved patient cohorts with multiple, mostly serious comorbidities, which substantially influence both treatment allocation and clinical outcomes. The absence of standardized methods to control for confounding factors, particularly comorbidity burden, represents a critical limitation across the studies. Consequently, the interpretation of comparative mortality data between surgical and conservative cohorts must be approached with caution. The current body of evidence highlights an urgent need for prospective, risk-adjusted studies to guide management and develop evidence-based, harmonized treatment algorithms.

Truumees et al. [64], reporting on the clinical characteristics of 96 consecutive patients presenting with hematogenous spinal osteomyelitis, found that the lumbar spine was the most commonly affected region (50%), followed by the thoracic spine (44%) and the cervical spine (17%). Similarly, in our meta-analysis, the lumbar spine was most frequently involved in HPSSI, with an average of 62.2% (range: 52.9–77.5%), followed by the thoracic spine at 27.7% (range: 19–34%) and the cervical spine at 14.9% (range: 3.5–22%), based on the affected spinal segments. In Truumees et al.’s study, 14.1% of the cases of infection included two or more spinal regions [64]. In 11 of 12 studies in our meta-analysis, infection involved >2 (2–4) spinal regions (cervical, thoracic, lumbar, lumbosacral). Variation in the spinal level(s) affected by infection has the potential to introduce selection bias in comparative analyses, potentially influencing outcome interpretation across studies.

In a previous meta-analysis, Thavarajasingam et al. [65] reported that early surgical intervention, compared to conservative management, was associated with a 39% relative risk reduction in mortality (*p* < 0.01). Similarly, two studies included in our meta-analysis provide some evidence suggesting that early surgical management may reduce mortality in selected patients with severe spinal infections [26,33].

Consistent with previous reports, comorbidity burden, frailty, poor functional status, and advanced age (Table 1) emerged as strong predictors of mortality [27,29,32,56]. Such factors may reflect underlying physiological vulnerability and an impaired capacity of the host to respond effectively to infection. Additionally, patients presenting with more severe infections—manifesting as neurological deficits, spinal instability, or medical treatment failure—were more frequently selected for surgical intervention, potentially introducing selection bias in comparative analyses. Surgical interventions included traditional open anterior, posterior, or combined approaches, as well as minimally invasive or percutaneous techniques. Percutaneous endoscopic surgery reliably decreases open approach-related complications and comorbidities in elderly and severely immune-compromised patients, offering safety and efficacy. Decompression of neural elements by stand-alone laminectomy, laminotomy, and debridement with/without spinal instrumentation for HPSSI has been described [47]. Stand-alone decompression (e.g., wide laminectomy) remains a valuable “damage-control” or emergency procedure, particularly for critically ill or hemodynamically unstable patients—most often those with abscess formation or severe comorbidities—who require urgent neural decompression due to progressive paralysis. This surgical technique is well established and can be performed safely even by less experienced surgeons. However, such emergency decompression is frequently associated with iatrogenic instability, which can increase the risk of complications, including infection relapse, persistent pain, and late-onset spinal instability. In contrast, combined decompression and stabilization is generally preferred for patients with infection-related vertebral body destruction, multilevel disease, or mechanical instability resulting from endplate erosion due to the infectious process. This approach facilitates earlier mobilization, reduces the risk of further neurological deterioration, and improves long-term spinal alignment. Nonetheless, surgical decisions should always be individualized, taking into account the extent of infection, degree of spinal instability, and the patient’s overall comorbidity profile.

In spite of this limitation, an increasing body of evidence indicates that early surgical intervention is associated with improved survival outcomes. Thavarajasingam et al. [65] demonstrated a 39% relative risk reduction in mortality with early surgery compared to conservative therapy, and similar findings were echoed by meta-analyses [33,35]. The international propensity-matched cohort by Neuhoff et al. [66] further reinforced this association, revealing significantly lower mortality among surgically treated patients (4%) versus those managed conservatively (24%, *p* < 0.001), even when neurological outcomes at discharge were comparable. These data collectively suggest that prompt surgical intervention, when appropriately indicated, may confer a survival advantage in selected high-risk patients.

In contrast, **recurrence rates** were low across studies and did not differ substantially between conservative and surgical treatments. Although comorbidities such as diabetes, malignancy, and immunosuppression were prevalent, their association with infection recurrence was not consistently observed [31]. Instead, disease-specific features—including paraspinal abscesses, multilevel involvement, and intraspinal abscess formation—were more closely associated with treatment failure or relapse [27,58]. However, these associations varied among studies, and some predictors—such as kyphotic progression after laminectomy without instrumentation—were attributable to structural issues rather than infectious processes [25].

The length of **hospital stays** demonstrated considerable heterogeneity across studies, influenced by patient selection, disease severity, and institutional practice patterns. Several analyses indicated that early surgical intervention reduced hospital stay by approximately 7–18 days compared to conservative management [32,33,65], whereas others observed no significant difference [58]. Comorbidities, frailty, and complications such as abscess formation, sepsis, and neurological compromise were associated with prolonged hospitalization regardless of treatment type.

Collectively, these findings highlight the multifactorial nature of outcomes in spinal infections. While surgery appears to improve survival and may reduce hospital stay in selected patients, baseline frailty, infection severity, and systemic comorbidities remain the primary determinants of prognosis.

Although current evidence regarding the optimal management of hematogenous primary spinal infections (HPSSI) remains inconclusive, our meta-analysis highlights the importance of individualized treatment strategies. Management decisions should be guided by the specific diagnosis, identification of causative pathogens through blood and tissue cultures, comorbidity burden, patient frailty, and neurological status. Early surgical consultation is warranted in cases of sepsis, progressive neurological compromise, or extensive abscess formation associated with neurological deterioration. Conversely, conservative therapy may be appropriate for clinically stable patients under close clinical and radiological monitoring. Adopting a multidisciplinary, risk-adapted approach may optimize survival, reduce neurological complications related to spinal cord compression, and improve functional outcomes—even in the absence of definitive evidence favoring a single therapeutic modality. Future standardized, prospective studies are essential to refine diagnostic and therapeutic strategies, particularly focusing on early and accurate detection using modern infection biomarkers, to establish evidence-based management guidelines for HPSSI.

### 4.1. Mortality Predictors in Hematogenous Spinal Infection

Jung et al. (2021) [27] identified several independent risk factors for mortality, including advanced age, multiple comorbidities, presence of secondary infectious foci, and neurological deficits. Similarly, Zadran et al. (2020) [29] developed a predictive model incorporating five variables associated with increased mortality: elevated Charlson Comorbidity Index (CCI), failure of C-reactive protein (CRP) normalization, cardiovascular disease, thoracic-level infection, and decreased Karnofsky performance score.

Frailty has also emerged as an important prognostic determinant. Alas et al. (2020) [32] demonstrated that an elevated Modified Frailty Index (mFI) correlated with increased short-term mortality, whereas operative intervention was associated with lower 30-day and 1-year mortality compared with conservative treatment. Collectively, these findings indicate that frailty and systemic comorbidities play a central role in determining outcomes and should be integrated into preoperative risk assessment and decision-making frameworks.

Several studies suggest that, when guided by standardized protocols, surgical management does not increase mortality compared with conservative approaches. Zadran et al. (2020) [29] reported comparable mortality rates between conservative and surgical groups when treatment followed consensus-based criteria. Importantly, surgical cohorts often include patients with complications, progressive deformity, or poor response to medical therapy—factors that inherently confer higher baseline risk.

Tsai-Tsoung-Ting (2017) [33] observed that early surgical intervention for pyogenic spondylodiscitis resulted in better prognosis, shorter hospitalization, and improved postoperative quality of life. Verla et al. (2020) [25] further emphasized the importance of stabilization and decompression in cases of thoracolumbar osteomyelitis/discitis with a risk of kyphotic progression, suggesting that timely surgical correction may prevent late deformity and secondary neurological deterioration.

### 4.2. Strategies to Reduce Mortality

Despite heterogeneous methodologies, several recurring themes emerge regarding mortality reduction in spinal infections. First, early multidisciplinary management—including coordinated input from spine surgeons, infectious disease specialists, and rehabilitation teams—appears pivotal. Jung et al. (2021) [27] proposed that lower relapse rates in certain cohorts might reflect intensified management, such as earlier radical debridement, combined antibiotic regimens in patients with implants, and specialist-directed care.

Second, systematic frailty and comorbidity assessment (e.g., via the Modified Frailty Index or Charlson Comorbidity Index) can identify high-risk patients who may benefit from preoperative optimization of nutritional, cardiovascular, and metabolic status.

Third, standardization of surgical indications and antibiotic protocols is necessary to minimize variability and improve comparability across studies. The implementation of unified, evidence-based guidelines—supported by prospective multicenter registries—would facilitate benchmarking of mortality outcomes and quality improvement initiatives.

Finally, targeted management of biomechanical sequelae such as infection-related kyphosis is essential. Structural instability not only contributes to pain and disability but may also exacerbate cardiopulmonary strain and infection recurrence, indirectly influencing mortality.

### 4.3. Study Limitations

Despite accumulating evidence, current studies on hematogenous primary spinal infections remain limited by methodological heterogeneity and retrospective design. Most available data are derived from single-center observational cohorts, introducing selection bias related to baseline comorbidities, infection severity, and treatment indication. Sample sizes were often small and underpowered, and reporting of key outcomes such as cause-specific mortality, recurrence, and complications was inconsistent. Variability in surgical indications, operative techniques, and follow-up duration further constrain comparison and generalizability across institutions.

Advanced age, frailty, multiple comorbidities, and neurological impairment are independent predictors of mortality in patients with HPSSI. All twelve studies included in this meta-analysis were retrospective; no randomized controlled trials (RCTs) were identified in the current literature addressing hematogenous spinal infection. However, only a few studies mentioned adjusting for confounding factors such as comorbidities. Most studies did not report methods for controlling for these confounding factors. As summarized in Table 1, nine of the twelve studies involved patient cohorts with serious and multiple comorbidities, which substantially influence both treatment allocation and clinical outcomes. The absence of standardized methods to control for confounding factors, particularly comorbidity burden, represents a critical limitation across most studies. Consequently, the interpretation of comparative mortality data between surgical and conservative cohorts must be approached with caution. The current body of evidence highlights an urgent need for prospective, risk-adjusted studies to guide management and develop evidence-based, harmonized treatment algorithms.

### 4.4. Future Directions

Future research should focus on identifying patient subgroups that derive the greatest survival or functional benefit from early surgical intervention. Prospective, multicenter studies or randomized controlled trials, where feasible, are needed to minimize bias and ensure adequate adjustment for confounding factors such as frailty and infection severity. Standardized diagnostic criteria, uniform outcome measures, and harmonized surgical indications would improve comparability among studies. Incorporating validated comorbidity indices, biomarkers of systemic infection, and advanced imaging parameters may facilitate the development of predictive models to guide individualized treatment decisions. Long-term functional, quality-of-life, and recurrence outcomes should also be systematically evaluated to capture the full clinical impact of both surgical and conservative management.

## 5. Conclusions

While some evidence suggests a potential short-term mortality benefit with surgical management of primary hematogenous spinal infections, the overall data remain heterogeneous and inconclusive. The lack of consistent reporting on key clinical outcomes, including recurrence and mortality stratified by treatment modality, underscores the need for high-quality, prospective studies to better inform clinical decision-making.

## Figures and Tables

**Table 1:** Common characteristics of the 12 studies included in the meta-analysis.

Paper	Single- or Multi-Center Study	Diagnosis	Conservative Group Pts	Surgical Group Pts	Median Age Years in Surg Group	Median Age Years in Cons Group	Gender (M, F)	Concomitant Diseases/Comorbidities	Causative Bacteria	Infection Location	Rate of Positive Cultures
Verla 2020 [25]	Single	Spinal Osteomyelitis/Discitis	12	8	51.7	51.7	(81 M−19 F)%	Diabetes, final-stage renal disease, intravenous drug users, sacral ulcers	MRSA (4 pts)	T10-L2	13/16 pts (81%)
Lener 2020 [26]	Single	Spondylodiscitis	51	146	65.1 ± 12.4	71.2 ± 9.6	(66.5 M−33.5 F)%	Renal failure, diabetes, dental disease	Multi-sensitive *S. aureus* (*n* = 5/24, 20.8%)	Cervical 24, thoracic 37, lumbar 116, cervical and thoracic 2, cervical and lumbar 1, thoracic and lumbar 17	68.6%
Jung 2021 [27]	Multicenter (2)	Vertebral Osteomyelitis (VO)	32	60	69 [56.5–74]	69 [56.5–74]	not reported	Diabetes mellitus, congestive heart failure, cardiovascular, peripheral vascular (arterial), cerebro-vascular, renal, liver, or chronic lung disease, rheumatoid arthritis	*S. aureus* only	Lumbosacral (67%)	63% (38/60)
Hasan 2021 [28]	Multicenter (5)	Pyogenic Spondylodiscitis	26	14	36.4 ± 11.8	36.4 ± 11.8	(20 M−80 F)%	Diabetes, hypertension, urinary tract infection, steroid intake, smoking	*S. aureus*, *E. coli*	Lumbar 31 (77.5%), cervical 8 (20%), thoracic 1 (2.5%)	Blood culture positive in 42.5%, biopsy culture positive in 82.5%
Behmanesh 2023 [30]	Single	Pyogenic Spinal Infection	55	155	67.3 ± 14.3	69.6 ± 16.2	(66 M−34 F)%	Not reported	*S. aureus*, *E. coli, Streptococcus*	Cervical 45 patients (40 vs. 5), thoracic 54 patients (38 vs. 16), lumbar 111 patients (77 vs. 34)	surg group 100 (65%) pts, cons group 7 (13%) pts
Zadran 2020 [29]	Single	Vertebral Osteomyelitis (VO)	50	75	67.25 (38–92)	67.24 (38–92)	(71 M−29 F)%	Diabetes, cardiovascular disease, lung disease, cancer, renal disease, intravenous drug abuse, alcoholism	*S. aureus*, *S. epidermitis*, *Streptococcus*, *E. coli*, *Enterococci*	Τhoracic: cons 13 (26%) vs. surg 22 (29%); non-thoracic: cons 37 (74%) vs. surg 53 (71%)	Not reported
Canouï 2019 [31]	Single	Spondylitis, Discitis, Spondylodiscitis, Osteomyelitis	62	28	64.3 (40–80)	64.4 (32–93)	(63 M−37 F)%	Diabetes mellitus, alcohol users, malignancy, heart disease, immunosuppression	*S. aureus* 19 (31%) vs. 13 (46%), coagulase-negative *Staphyloccocus* 8 (13%) vs. 2 (7%), *Streptococci* 19 (31%) vs. 8 (29%), *Enterobacteriaceae* 15 (24%) vs. 5 (18%)	Cervical: cons 13 (21%) vs. surg 7 (25%), thoracic: cons 21 (34%) vs. surg 9 (32%), lumbosacral: cons 34 (55%) vs. surg 12 (43%)	Not reported
Alas 2020 [32]	Multicenter	Spondylodiscitis	73	43	62.9 ± 9.7	70.7 ± 12.7	Not reported	Diabetes, chronic renal failure, neoplasia, chronic alcoholism, intravenous drug use, rheumatological disease, chronic steroid use, chronic immunosuppression, liver cirrhosis	*S. aureus*, *MRSA*, *Klebsiella pneumoniae*, *E. coli*	Lumbar (57.8%), thoracic (19.0%), cervical (14.7%)	90.5%
Tsai Tsung-Ting 2017 [33]	Single	Pyogenic Spondylodiscitis	47	43	58.9 (16–82)	62.5 (30–85)	(68 M−32 F)%	Diabetes, end-stage renal disease, liver cirrhosis, rheumatoid arthritis, drug addiction	Not reported	Not reported	cons: 23 (48.9%), surg: 36 (83.7%)
Yoshimoto 2011 [56]	Single	Pyogenic Spondylitis	30	15	71 (65–93)	71 (65–93)	(62 M−38 F)%	Diabetes, malignant tumors, pyelonephritis, renal failure, esophageal rupture, chronic hepatitis	*S. aureus* in 13 cases (*MRSA* 9/13)	Cervical 5, thoracic 8, thoracolumbar 6, lumbar 26	36%
Woertgen 2006 [57]	Single	Pyogenic Spinal Infection	28	34	64.3 (32–82)	64.3 (32–82)	(56 M−44 F)%	Not reported	*S. aureus*	Lumbar (59.6%), thoracic and thoracolumbar (31%), cervical (9.6%)	49/62 (79%)
Xie B-L 2024 [58]	Multicenter (3)	Pyogenic Spondylitis	53	59	60.82 ± 11.50	60.82 ± 11.50	(61 M−39 F)%	Not reported	*S. aureus*, *E. coli*, *S. epidermitis*	Cervical 2 (3.8) vs. 1 (1.7), thoracic 10 (18.9) vs. 9 (15.3), lumbar 34 vs. 37, cervicothoracic 0 vs. 0, thoracolumbar 1 (1.9) vs. 2 (3.4), lumbosacral 6 (11.3)	cons 39.4% (13/33), surg 32.7% (16/49)

**Table 2:** Newcastle–Ottawa scores for the 12 retrospective studies included in the meta-analysis.

Author	Publication	Selection	Comparability	Outcome	Total
Caoui [31]	2019	4	2	3	**9**
Hasan [28]	2021	3	1	3	**7**
Jung [27]	2021	4	2	3	**9**
Lener [26]	2020	4	2	3	**9**
Yoshimoto [56]	2010	3	0	3	**6**
Tsai [33]	2017	3	1	2	**6**
Verla [25]	2020	4	1	2	**7**
Woertgen [57]	2006	3	1	2	**6**
Zadran [29]	2020	4	1	3	**8**
Alas [32]	2020	4	1	3	**8**
Bang-lin Xie [58]	2024	4	1	3	**8**
Behmanesh [30]	2021	3	0	2	**5**
**Average**		**3.58/4**	**1.08/2**	**2.66/3**	**7.33/9**

**Table 3:** Mortality outcomes.

Study	Mortality Rate	Follow-Up Duration	Patient Characteristics
Alas et al., 2020 [32]	Surgical: 2.3% (30 days), 11.6% (1 year); Conservative: 17.8% (30 days), 20.5% (1 year)	30 days, 1 year	Adults, mean age 67.8 y, spondylodiscitis
Lener et al., 2020 [26]	Surgical: 8/146 (6%); Conservative: 9/51 (18%)	2010–2017	Mean age 65–71 y, spinal infection
Canouï et al., 2019 [31]	Surgical: 3/28 (11%); Conservative: 7/62 (11%)	12 months	Mean age 64 y, 63% male, hematogenous pyogenic vertebral osteomyelitis
Tsai et al., 2017 [33]	9 deaths (7 conservative, 2 surgical), all excluded from main analysis	During hospitalization	Mean age 60.7 y, 68% male
Behmanesh et al., 2023 [30]	Surgical: 37/155 (24%); Conservative: 13/55 (24%)	Last follow-up	Mean age 68.6 y, 66% male

**Table 4:** Recurrence outcomes.

Study	Conservative Group Recurrence	Surgical Group Recurrence	Statistical Significance
Canouï et al., 2019 [31]	5% (total 4/90; group-specific, no mention found)	5% (total 4/90; group-specific, no mention found)	*p* = 1.000
BL Xie et al., 2024 [58]	9/53 (17.0%)	7/59 (11.8%)	*p* = 0.440
Alas et al., 2020 [32]	16.4% (no numerical data reported)	11.6% (no numerical data reported)	*p* = 0.592

**Table 5:** Hospital stay outcomes.

Study	Surgical Length of Stay (Avg)	Conservative Length of Stay (Avg)	Statistical Significance
BL Xie et al., 2024 [58]	25.02 ± 10.24 days	23.55 ± 10.86 days	No significant difference
Tsai et al., 2017 [33]	33.4 (SD = 17.5) days	51.2 (SD = 23.2) days	*p* < 0.001
Alas et al., 2020 [32]	23.9 ± 18.2 days	40.5 ± 30.7 days	*p* = 0.002

## Data Availability

The data supporting this meta-analysis have been deposited in the Mendeley Data repository and can be accessed at DOI: 10.17632/4w88x9t75k.1.

## Supplementary Materials

The following supporting information can be downloaded at https://www.mdpi.com/article/10.3390/jcm14248650/s1. File S1: ROBINS-I Assessment—Body-level and Study-level Judgments; File S2: GRADE Summary of Findings Table; File S3: Plain Language Summary in Cochrane style format; File S4: PRISMA 2020 Checklist.

## Abbreviations

The following abbreviations are used in this manuscript:

HPSSI	Hematogenous Primary Septic Spinal Infection
VO	Vertebral Osteomyelitis
