# Supplementary material for: Is Conservative Treatment Superior to Surgical Intervention in Hematogenous Primary Septic Spinal Infection in Terms of Mortality, Recurrence, and Hospital Stay? A Systematic Review and Meta-Analysis
Source: J Clin Med. 2025 Dec 6;14(24):8650. doi: 10.3390/jcm14248650 (PMC12734290; doi:10.3390/jcm14248650)
Supplement: Supplementary file 1 [file jcm-14-08650-s001.zip › File S1-ROBINS_I_Assessment_Study_Level.pdf]

ROBINS-I Assessment — Body-level and Study-level Judgments

A. Overall body-level ROBINS-I judgments

Summary: Overall judgment: Serious risk of bias

Domain-level judgments (overall body of evidence):

ROBINS-I Domain	Judgment / Rationale (short)
Bias due to confounding	Serious — retrospective cohorts, confounding by indication and inconsistent adjustment across studies.
Bias in selection of participants	Serious — retrospective recruitment, variable exclusions, differential referral/triage and some excluded early deaths.
Bias in classification of interventions	Serious — heterogeneous definitions of 'surgery' vs 'conservative', variable timing and techniques.
Bias due to deviations from intended interventions	Moderate — crossovers (conservative to surgery) and inconsistent handling reported.
Bias due to missing data	Moderate — variable follow-up completeness; some studies excluded early deaths or had incomplete outcome reporting.
Bias in measurement of outcomes	Moderate — mortality objective, recurrence variably defined, hospital stay affected by local practice.
Bias in selection of the reported result	Moderate — selective outcome reporting plausible; small numbers for some outcomes reduce reliability.

B. Study-level ROBINS-I judgments (12 included retrospective comparative studies)

Study	NOS	Confounding	Selection	Classification	Deviations	Missing data	Measurement	Reporting	Overall
Verla 2020	7	Serious	Serious	Moderate to Serious	Moderate	Moderate	Moderate	Moderate	Serious
Lener 2020	9	Moderate	Moderate	Moderate	Moderate	Low to Moderate	Low to Moderate	Low to Moderate	Moderate
Jung 2021	9	Moderate	Moderate	Moderate	Moderate	Low to Moderate	Low to Moderate	Low to Moderate	Moderate
Hasan/ Hassan 2021	7	Serious	Serious	Moderate to Serious	Moderate	Moderate	Moderate	Moderate	Serious
Behmanesh 2021	5	Serious	Serious	Serious	Serious	Serious	Serious	Serious	Serious
Zadran 2019	8	Moderate	Moderate	Moderate	Moderate	Low to Moderate	Low to Moderate	Low to Moderate	Moderate
Canouï 2019	9	Moderate	Moderate	Moderate	Moderate	Low to Moderate	Low to Moderate	Low to Moderate	Moderate
Alas 2020	8	Moderate	Moderate	Moderate	Moderate	Low to Moderate	Low to Moderate	Low to Moderate	Moderate
Tsai Tsung-Ting 2017	6	Serious	Serious	Serious	Moderate	Moderate	Moderate	Moderate	Serious
Yoshimoto 2010	6	Serious	Serious	Serious	Moderate	Moderate	Moderate	Moderate	Serious
Woertgen 2006	6	Serious	Serious	Serious	Moderate	Moderate	Moderate	Moderate	Serious
Xie B-L 2024	8	Moderate	Moderate	Moderate	Moderate	Low to Moderate	Low to Moderate	Low to Moderate	Moderate

C. Short rationales and notes (per study)

Verla 2020 (NOS ≈ 7):
Confounding: Serious; Selection: Serious; Classification: Moderate to Serious; Deviations: Moderate; Missing data: Moderate; Measurement: Moderate; Reporting: Moderate. Overall: Serious.

Rationale: Best-effort judgement based on manuscript details (retrospective design, reported baseline imbalances, single vs multi-center status, and NOS score).

Lener 2020 (NOS ≈ 9):
Confounding: Moderate; Selection: Moderate; Classification: Moderate; Deviations: Moderate; Missing data: Low to Moderate; Measurement: Low to Moderate; Reporting: Low to Moderate. Overall: Moderate.

Rationale: Best-effort judgement based on manuscript details (retrospective design, reported baseline imbalances, single vs multi-center status, and NOS score).

Jung 2021 (NOS ≈ 9):
Confounding: Moderate; Selection: Moderate; Classification: Moderate; Deviations: Moderate; Missing data: Low to Moderate; Measurement: Low to Moderate; Reporting: Low to Moderate. Overall: Moderate.

Rationale: Best-effort judgement based on manuscript details (retrospective design, reported baseline imbalances, single vs multi-center status, and NOS score).

Hasan/ Hassan 2021 (NOS ≈ 7):
Confounding: Serious; Selection: Serious; Classification: Moderate to Serious; Deviations: Moderate; Missing data: Moderate; Measurement: Moderate; Reporting: Moderate. Overall: Serious.

Rationale: Best-effort judgement based on manuscript details (retrospective design, reported baseline imbalances, single vs multi-center status, and NOS score).

Behmanesh 2021 (NOS ≈ 5):
Confounding: Serious; Selection: Serious; Classification: Serious; Deviations: Serious; Missing data: Serious; Measurement: Serious; Reporting: Serious. Overall: Serious.

Rationale: Best-effort judgement based on manuscript details (retrospective design, reported baseline imbalances, single vs multi-center status, and NOS score).

Zadran 2019 (NOS ≈ 8):
Confounding: Moderate; Selection: Moderate; Classification: Moderate; Deviations: Moderate; Missing data: Low to Moderate; Measurement: Low to Moderate; Reporting: Low to Moderate. Overall: Moderate.

Rationale: Best-effort judgement based on manuscript details (retrospective design, reported baseline imbalances, single vs multi-center status, and NOS score).

Canouï 2019 (NOS ≈ 9):
Confounding: Moderate; Selection: Moderate; Classification: Moderate; Deviations: Moderate; Missing data: Low to Moderate; Measurement: Low to Moderate; Reporting: Low to Moderate. Overall: Moderate.

Rationale: Best-effort judgement based on manuscript details (retrospective design, reported baseline imbalances, single vs multi-center status, and NOS score).

Alas 2020 (NOS ≈ 8):
Confounding: Moderate; Selection: Moderate; Classification: Moderate; Deviations: Moderate; Missing data: Low to Moderate; Measurement: Low to Moderate; Reporting: Low to Moderate. Overall: Moderate.

Rationale: Best-effort judgement based on manuscript details (retrospective design, reported baseline imbalances, single vs multi-center status, and NOS score).

Tsai Tsung-Ting 2017 (NOS ≈ 6):
Confounding: Serious; Selection: Serious; Classification: Serious; Deviations: Moderate; Missing data: Moderate; Measurement: Moderate; Reporting: Moderate. Overall: Serious.

Rationale: Best-effort judgement based on manuscript details (retrospective design, reported baseline imbalances, single vs multi-center status, and NOS score).

Yoshimoto 2010 (NOS ≈ 6):
Confounding: Serious; Selection: Serious; Classification: Serious; Deviations: Moderate; Missing data: Moderate; Measurement: Moderate; Reporting: Moderate. Overall: Serious.

Rationale: Best-effort judgement based on manuscript details (retrospective design, reported baseline imbalances, single vs multi-center status, and NOS score).

Woertgen 2006 (NOS ≈ 6):
Confounding: Serious; Selection: Serious; Classification: Serious; Deviations: Moderate; Missing data: Moderate; Measurement: Moderate; Reporting: Moderate. Overall: Serious.

Rationale: Best-effort judgement based on manuscript details (retrospective design, reported baseline imbalances, single vs multi-center status, and NOS score).

Xie B-L 2024 (NOS ≈ 8):
Confounding: Moderate; Selection: Moderate; Classification: Moderate; Deviations: Moderate; Missing data: Low to Moderate; Measurement: Low to Moderate; Reporting: Low to Moderate. Overall: Moderate.

Rationale: Best-effort judgement based on manuscript details (retrospective design, reported baseline imbalances, single vs multi-center status, and NOS score).
