# Supplementary material for: Is Conservative Treatment Superior to Surgical Intervention in Hematogenous Primary Septic Spinal Infection in Terms of Mortality, Recurrence, and Hospital Stay? A Systematic Review and Meta-Analysis
Source: J Clin Med. 2025 Dec 6;14(24):8650. doi: 10.3390/jcm14248650 (PMC12734290; doi:10.3390/jcm14248650)
Supplement: Supplementary file 1 [file jcm-14-08650-s001.zip › File S2-GRADE_SoF_Table.pdf]

GRADE Summary of Findings Table

Outcome	Studies / Patients	Relative Effect	Absolute Effect (examples)	Certainty (GRADE)
Mortality	5 studies (subset of 12)	OR 0.82 (95% CI 0.30–2.18); I^2=74%	If baseline 10% → 8% (3–19%) If baseline 20% → 17% (6–35%)	Very low
Infection Recurrence	3 studies	OR 1.49 (95% CI 0.32–6.95); I^2=73%	If baseline 10% → 14% (3–41%) If baseline 20% → 27% (6–63%)	Very low
Length of Hospital Stay	3 studies	MD −10.56 days (95% CI −24.90 to +3.97); I^2=92%	Surgery may shorten stay by ~11 days, but CI includes no diff.	Very low

Footnotes

1. Mortality: Retrospective cohort design; serious risk of bias; high inconsistency; imprecise estimate.

2. Recurrence: Retrospective studies; variable outcome definitions; high heterogeneity; very imprecise estimate.

3. Hospital Stay: Serious risk of bias; very high heterogeneity; imprecise pooled estimate.
