# Supplementary material for: Is Conservative Treatment Superior to Surgical Intervention in Hematogenous Primary Septic Spinal Infection in Terms of Mortality, Recurrence, and Hospital Stay? A Systematic Review and Meta-Analysis
Source: J Clin Med. 2025 Dec 6;14(24):8650. doi: 10.3390/jcm14248650 (PMC12734290; doi:10.3390/jcm14248650)
Supplement: Supplementary file 1 [file jcm-14-08650-s001.zip › File S3-Plain Language Summary in Cochrane style format.pdf]

Plain Language Summary in Cochrane style format

Background

Hematogenous Primary Septic Spinal Infection (HPSSI) is a serious bacterial infection of the spine that can cause severe pain, neurological deficits even paralysis if left untreated. It most often affects older ages and immune suppressed patients. Medical doctors treat HPSSI either with **conservative management**—antibiotics, bed rest, and bracing—or **surgery** to remove infected tissue and stabilization of the spine. However, it remains unclear which approach leads to better outcomes in terms of **survival, infection recurrence and length of hospital stay**.

What did the researchers do?

The authors conducted a **systematic review and meta-analysis**, which means they carefully collected and analyzed data from previously published studies. They searched four major scientific databases—PubMed, Cochrane, ScienceDirect, and Scopus—for studies comparing surgical and conservative treatment in adults with HPSSI (not caused by tuberculosis, fungi, or previous surgery).
A total of **twelve studies** including **1,199 patients** were analyzed. The main outcome measured was **mortality (death rate)**, while secondary outcomes included **infection recurrence** and **hospital stay duration**.

What did they find?

The results showed that there was **no significant difference in overall mortality** between surgical and conservative treatments. Some studies reported lower short-term death rates among patients who had surgery, but others found no difference at all. Infection recurrence rates were generally low (around 5–16%) and did not differ meaningfully between treatment types. Hospital stay lengths also varied widely: some studies showed shorter stays for surgical patients, while others found no difference. Across all studies, **older age, frailty, and multiple health problems** were the most consistent factors linked to worse outcomes, regardless of treatment. The authors also noted that differences in study design and patient characteristics made it difficult to draw firm conclusions.

What does it mean?

This meta-analysis suggests that neither surgery nor conservative treatment is clearly superior for all patients with HPSSI. While surgery may help some patients—especially those with severe infections or nerve compression—the overall evidence remains **inconclusive**. The study highlights the need for **future high-quality clinical trials** with standardized definitions and patient criteria to better determine who benefits most from each treatment.
Early diagnosis, accurate identification of the bacteria causing infection, and timely treatment—whether surgical or conservative—remain essential for improving patient outcomes and reducing complications such as recurrence, prolonged hospital stay, or death.
